# Supplementary material for: Dissection of specific binding of HIV-1 Gag to the 'packaging signal' in viral RNA
Source: eLife. 2017 Jul 20;6:e27055. doi: 10.7554/eLife.27055 (PMC5531834; doi:10.7554/eLife.27055)
Supplement: Supplementary file 1. — DOI: http://dx.doi.org/10.7554/eLife.27055.024 [file elife-27055-supp1.docx]

| **Supplementary file 1.** Diffusion coefficients of the dye used to calibrate the FCS setup and the ***D*** of Lysozyme labeled with AF647 | | | | | | |
| --- | --- | --- | --- | --- | --- | --- |
| **Dye** | **Reported *D* of the dye (μm^2^/s)** | **Source of the reported diffusion coefficient of the dye** | ***Estimated ω_xy_ (*μ*m)*** | **Estimated *D* of lysozyme (μm^2^/s)** | **Reported *D* of lysozyme# (μm^2^/s)** | **%Error** |
| AF647 | 300 | Optics Express 20(27):28379-29387 (2012) | 0.32 | 122 | 112 | 9 |
| AF647 | 330 | PicoQuant* | 0.33 | 135 |  | 21 |
| Note: * www.picoquant.com/images/uploads/page/files/7353/appnote_diffusioncoefficients.pdf and #Van Holde, Johnson and Ho. Principles of Physical Biochemistry 2nd edition | | | | | | |
|  |  |  |  |  |  |  |

**Supplementary file 1.** Conversion of the diffusion time into a diffusion coefficient requires estimation of the size of the confocal volume (see the Fluorescence correlation spectroscopy and data analysis section). To calculate the size of the confocal volume the dimensions of the laser in the *x,y*-plane (***ω_xy_*** in μm) must be obtained by measuring the diffusion time of a fluorescent molecule with a known diffusion coefficient (*i.e.,* AlexaFluor 647 or AF647). However, there are two reported values for this dye. To determine the most accurate diffusion coefficient of AF647 we measured the diffusion coefficient of fluorescently labeled lysozyme and used both reported values of AF647 to determine the ***D*** of this protein (“estimated ***D***”). The estimated ***D*** was compared with the diffusion coefficient that has been reported elswhere. Using a diffusion coefficient for AF647 of 300 μm^2^/s gave the best match to the reported diffusion coefficient of lysozyme.
